# Supplementary material for: Poliovirus excretion among children with primary immune deficiency in Pakistan: a pilot surveillance study protocol
Source: BMJ Open. 2021 Jul 28;11(7):e045904. doi: 10.1136/bmjopen-2020-045904 (PMC8319991; doi:10.1136/bmjopen-2020-045904)
Supplement: Author's manuscript [file bmjopen-2020-045904.draft_revisions.pdf]

BMJ Open is committed to open peer review. As part of this commitment we make the peer review history of every article we publish publicly available.

When an article is published we post the peer reviewers' comments and the authors' responses online. We also post the versions of the paper that were used during peer review. These are the versions that the peer review comments apply to.

The versions of the paper that follow are the versions that were submitted during the peer review process. They are not the versions of record or the final published versions. They should not be cited or distributed as the published version of this manuscript.

BMJ Open is an open access journal and the full, final, typeset and author-corrected version of record of the manuscript is available on our site with no access controls, subscription charges or pay-per-view fees (http://bmjopen.bmj.com).

If you have any questions on BMJ Open's open peer review process please email info.bmjopen@bmj.com

BMJ Open

Poliovirus Excretion Among Children with Primary Immune Deficiency in Pakistan- A Pilot Surveillance Study Protocol

Journal:	*BMJ Open*
Manuscript ID	bmjopen-2020-045904
Article Type:	Protocol
Date Submitted by the Author:	15-Oct-2020
Complete List of Authors:	Pethani, Asma; Aga Khan University Hospital, Pediatrics and Child Health<br>Kazi, Zaubina; Aga Khan University, Pediatrics and Child Health<br>Nayyar, Ujala; World Health Organization Country Office for Pakistan, Polio Eradication Initiative<br>Shafiq-ur-Rehman, Muhammad ; World Health Organization Country Office for Pakistan<br>Yousafzai, Muhammad Tahir; Aga Khan University, Pediatrics and Child Health<br>Ondrej, Mach; World Health Organization<br>Saleem, Ali; Aga Khan University, Paediatrics and Child Health
Keywords:	INFECTIOUS DISEASES, PAEDIATRICS, Public health < INFECTIOUS DISEASES

SCHOLARONE™
Manuscripts

I, the Submitting Author has the right to grant and does grant on behalf of all authors of the Work (as defined in the below author licence), an exclusive licence and/or a non-exclusive licence for contributions from authors who are: i) UK Crown employees; ii) where BMJ has agreed a CC-BY licence shall apply, and/or iii) in accordance with the terms applicable for US Federal Government officers or employees acting as part of their official duties; on a worldwide, perpetual, irrevocable, royalty-free basis to BMJ Publishing Group Ltd ("BMJ") its licensees and where the relevant Journal is co-owned by BMJ to the co-owners of the Journal, to publish the Work in this journal and any other BMJ products and to exploit all rights, as set out in our licence.

The Submitting Author accepts and understands that any supply made under these terms is made by BMJ to the Submitting Author unless you are acting as an employee on behalf of your employer or a postgraduate student of an affiliated institution which is paying any applicable article publishing charge ("APC") for Open Access articles. Where the Submitting Author wishes to make the Work available on an Open Access basis (and intends to pay the relevant APC), the terms of reuse of such Open Access shall be governed by a Creative Commons licence – details of these licences and which Creative Commons licence will apply to this Work are set out in our licence referred to above.

Other than as permitted in any relevant BMJ Author's Self Archiving Policies, I confirm this Work has not been accepted for publication elsewhere, is not being considered for publication elsewhere and does not duplicate material already published. I confirm all authors consent to publication of this Work and authorise the granting of this licence.

For peer review only

Poliovirus Excretion among Children with Primary Immune Deficiency in Pakistan, a Pilot Surveillance Study Protocol

Asma Pethani[1], Zaubina Kazi[1], Ujala Nayyar[2], Muhammad Shafiq-ur-Rehman[3], Muhammad Tahir Yousafzai[1], Mach Ondrej[4], Ali Faisal Saleem[1]*

[1]Department of Paediatrics & Child Health, Aga Khan University, Karachi

[2]Polio Eradication Initiative, World Health Organization, Pakistan

[3]World Health Organization and National Emergency Operations Centre, Pakistan

[4]World Health Organization, Geneva, Switzerland

*Corresponding author, ali.saleem@aku.edu

Abstract

Introduction:

Children with primary immunodeficiency disorders (PID) are more susceptible to developing viral infections and are at a substantially increased risk of developing paralytic poliomyelitis. Such children, if given oral polio vaccines tend to excrete poliovirus chronically that may lead to the propagation of highly divergent vaccine-derived poliovirus (VDPVs). Consequently, they may act as a reservoir for the community by introducing an altered virus potentially imposing a risk to global polio eradication. However, the risks of chronic and prolonged excretion are not well characterized in the study context. This study seeks to establish a pilot surveillance system for successful identification and monitoring of vaccine-derived poliovirus excretion among children with PID. It will assess whether the Jeffrey Modell warning signs of PID can be utilized as an appropriate screening tool for PID in Pakistan.

Methods and Analysis:

In this pilot surveillance, recruitment of PID cases is currently done at participating hospitals in Pakistan. Potential children are screened and tested against the Jeffery Model Foundation (JMF) signs for immunodeficiency and their stool is collected to test for poliovirus excretion. Cases excreting poliovirus are followed until the two consecutive negative stool samples are obtained over a period of six months. The data will be analyzed to calculate hospital-based proportions of total iVDPV cases over a two-year period and to determine the sensitivity and specificity of the JMF signs.

Ethics and Dissemination:

This protocol was reviewed and approved by the World Health Organization (WHO Reference-2018/811124-0), Aga Khan University (AKU ERC- 2018-0380-1029) and National Bioethics Committee (Ref No. 4-87 NBC-308-Y2). The results will be published in an open access peer-reviewed scientific journal and presented to the iVDPV working-group members, policy-makers,

For peer review only - http://bmjopen.bmj.com/site/about/guidelines.xhtml

paediatric consultants and fellow researchers with the same domain interest. It may be presented in scientific conferences and seminars in the form of oral or poster presentations.

**Strengths and limitations:**
• The study will provide evidence about the proportion of children categorized with iVDPV<br>• Future policy formulation of diseases not suitable for Oral Polio Vaccine administration with reference to the study data<br>• Unique surveillance network of Pediatricians across Pakistan.<br>• Areas having only secondary care hospitals may have under-reporting of PID cases

For peer review only

Introduction

Polio is a highly infectious viral disease which invades the nervous system of a child and may cause irreversible paralysis (1). The use of the live oral poliovirus vaccine (OPV) in routine and supplementary immunization activities (SIAs) has significantly reduced the incidence of paralytic poliomyelitis and transmission of wild polioviruses around the globe. This success can be attributed to the ability of this vaccine to induce mucosal immunity as well as serum neutralizing antibodies protective against paralytic poliomyelitis (2). However, individuals with primary immune deficiency disorders (PID) exposed to OPV through vaccination or household contacts are not only at increased risk of vaccine-associated paralytic poliomyelitis (VAPP) but may also have prolonged excretion of Sabin vaccine-derived polioviruses (VDPVs) (3). Sabin polioviruses are known to have increased transmissibility and neurovirulence following prolonged intestinal replication. Such individuals could theoretically transmit the virus to contacts and the general population following the interruption of wild poliovirus transmission (4). These polioviruses are called iVDPVs, which indicates that these viruses have replicated and evolved in immuno-compromised individuals. Previous studies in Sri Lanka, Egypt, Bangladesh and elsewhere demonstrated that it is essential to identify PID persons excreting polioviruses (1, 5, 6).

Poliovirus excretion into the environment negates global poliovirus eradication efforts. PID individuals infected with poliovirus are at risk of developing paralysis. Antiviral therapy will be offered to those identified immunodeficient children who are excreting polioviruses to prevent the spread of these polioviruses.

Aim:

Identification and diagnosis of PID is complex and requires specialist interventions. The Jeffrey Modell Foundation has developed 10 warning signs for the screening of suspected primary immunodeficiency patients (7). Although this tool has been used in various countries, its sensitivity and specificity in low-resource countries such as Pakistan has not been assessed. Therefore, the primary aim of this project is to develop a pilot surveillance system for the identification of primary immunodeficient children in sentinel sites of Pakistan which will provide the basis for the development of longer-term surveillance structure for the successful identification, genetic characterization and monitoring of vaccine-derived poliovirus excretion. Secondary aims will be to calculate the hospital-based proportion of iVDPV cases and to document the identified iVDPV cases in the WHO global registry list. Finally, it will help assess whether the Jeffrey Modell warning signs of PID are an appropriate screening tool for PID in Pakistan.

Methodology

Project Structure:

The surveillance of iVDPV is currently being undertaken at participatory tertiary healthcare facilities around Pakistan. This pilot surveillance will help in structuring a sustainable, long-term surveillance

For peer review only - http://bmjopen.bmj.com/site/about/guidelines.xhtml

for PID children in Pakistan. In the initial stage, mapping was executed to identify participatory hospitals and their Consultant Pediatricians. Master training workshops were then conducted for these paediatricians from the selected hospitals of each province of Pakistan who agreed to participate in iVDPV surveillance. The project was rolled out as follows:

- First 6 months: AKU Hospital + 3 other major tertiary care hospitals in Karachi
- Months 7-24: AKU Hospital + Major hospitals in Karachi + Tertiary hospitals located in the provinces of Sindh (Hyderabad), Punjab (Lahore, Multan, Islamabad), Balochistan (Quetta), Khyber Pakhtunkhwa and Gilgit-Baltistan.

Training of Master Trainers Workshop:

The master training workshops were conducted all over the country in collaboration with World Health Organization, Pakistan. All tertiary care hospitals in each province with paediatricians were approached. Across Pakistan, 49 tertiary care hospitals have been participating in iVDPV surveillance. The participatory rate from the province Sindh (n=7) and Balochistan (n=3) is 100%, followed by 72.5% (n=29) from the province Punjab. The participatory proportion from the province Khyber Pakhtunkhwa (n=10) is 38.5%, which is the lowest amongst the remaining provinces **(Refer to *Figure 1- Participating Tertiary Care Hospitals*.).** The purpose of the training workshop was to educate the master trainers who in turn guided their departmental doctors for screening and identification of PID children using the JMF tool.

The master training workshops were conducted by the principal investigator of this project who is a pediatric infectious diseases consultant at the Aga Khan University Hospital in collaboration with WHO experts in Polio Surveillance.

Co-ordination Process:

This study requires close collaboration and coordination amongst the stakeholders. The Aga Khan University (Pakistan campus) is the main coordination center for the study. A study coordinator serves as a liaison between the participatory hospitals in each province and the WHO provincial team, which collects the stool samples of identified PID children. National Institute of Health, Islamabad virology lab processes the stool samples and identifies the type of poliovirus.

Data Collection Process:

Children are identified by the paediatricians trained on Jeffrey Model Foundation warning signs and the diagnosis of primary immunodeficiency. Any child (up to 18 years of age) fulfilling the criteria of 2 or more positive JMF signs, will be further assessed and tested for HIV. The children who test negative for HIV, either suspected or confirmed PIDs are considered potential iVDPV excreters and are enrolled in the study. Enrolled children will be further tested for complete blood count (CBC), flow cytometry and quantitative measurement of immunoglobulins level (QIGs) after consulting with the co-ordination cell at the Aga Khan University. A consultant haematologist at the Aga Khan

For peer review only

University will review the flow cytometry and immunoglobulins level reports. After enrollment, WHO provincial teams collect two consecutive stool samples, which are examined for poliovirus at NIH Islamabad-Pakistan using standard poliovirus detection methodology used in the Global Polio Laboratory Network. The second stool sample will be collected 24 hours after the first sample. Any child found to be positive will be followed-up with monthly stool samples for 6 months or till two subsequent samples become negative. All iVDPVs detected will be documented in the WHO global registry list of known iVDPV cases.

Follow-up with the iVDPV cases:

The parents or guardians of iVDPV positive children are informed and followed up closely by EPI and Union council teams along with WHO provincial teams. Children are followed up until two consecutive negative stool samples for a period of six months. However, if the child is still found to be an excreter at 6 months, they will be provided anti-viral therapy for poliovirus excretion. This therapy would be provided to the affected child free of cost. The children who test negative for VDPVs or Sabin like virus, stool sample collection will be repeated after a year.

Process:

In order to maximize the surveillance coverage of primary immune deficient children, hospital pediatricians will need to be sufficiently sensitized to the necessary referral process.

Children with suspected PID screened using JMF warning signs will be enrolled and the following laboratory investigations would be sent for confirmation ***(Refer to Figure 2- Flow diagram of study process).***

1. Complete blood count (Absolute neutrophil and lymphocyte count)
2. HIV
3. Flow cytometry (B and T cell markers)
4. Quantitative immunoglobulins test (QIG)

Patient and Public Involvement:

Considering the nature of the study, it was not possible to have a priori patient involvement. The country leadership from World Health Organization and National Emergency Operations Centre (NEOC) were consulted during the study designing to safeguard public interest and safety.

Biological sample collection, transport and laboratory processing:

Blood Samples:

For peer review only

HIV:

For HIV testing, 3-5 ml of whole blood will be collected in a gel tube and K2-EDTA tube. Gel tube will be utilized for serology whereas, EDTA tube would use for PCR. Samples would be transported to the laboratory at 2-8 ^{0}C. Initial HIV screening will be performed by chemiluminescent immunoassay (CLIA) using ADVIA Centaur HIV Ag/Ab Combo assay kit run on ADVIA Centaur XP/CP analyzer. Positive cases will be further confirmed by immunochromatographic antibody detection on Bio-Rad Genius HIV 1 / 2 assay. In case of indeterminate results or discrepant results between the above two methods final confirmation will be made by HIV PCR on Xpert HIV-1 Qual Assay kit and GeneXpert Dx System.

CBC

For complete blood count, 3-4ml whole blood sample will be collected in tubes containing K2 EDTA as an anticoagulant. Samples will be transported as soon as possible to the stat/main laboratory. The temperature of 18 –25 ^{0}C will be maintained if the expected delivery time is within 8 hours, whereas 2 – 8 ^{0}C will be maintained if samples are delivered after 8 hours up to a maximum of 3 days. Samples will be run on the fully automated Sysmex XN series analyzers (Sysmex Japan).

Immunoglobulins

For immunoglobulin measurement, 3-5 ml of whole blood will be collected in a plain tube or gel tube. Transportation temperature of 18-25°C (for 8 Hours) and 2-8 °C after 8 hours to 24 hours will be maintained. Serum Immunoglobulin A, G and M (IgA, IgG, IgM) will be measured on ADVIA 1800 Chemistry analyzer (Siemens Healthcare Diagnostics Inc, NY, US).

Flow Cytometry

For lymphocyte subset analysis, 3-4ml of whole blood will be collected in tubes containing K2 EDTA or sodium heparin as an anticoagulant. All samples will be transported at 2-8^{0}C within 24 hours. Lymphocyte subset analysis will be performed by flow cytometry using FC500 MCL Flow Cytometer (Beckman Coulter, Miami, FL, USA). Monoclonal antibodies against following antigens were used: T-lineage-associated antigens CD3, CD4 and CD8; B-lineage antigen CD19 and Natural Killer cell antigen CD56.

For CBC and immunoglobulin levels, three different levels of manufacturer-provided controls (low, normal and high) will be run with each batch as an internal quality control measure. Whereas, for lymphocyte subset analysis 2 levels (low and normal) commercial controls will be used. External proficiency is assured by simultaneously analyzing samples from the College of American Pathologists 2 times per year for immunoglobulin levels and three times per year for both complete blood counts and lymphocyte subset analysis.

Following samples will be considered unsatisfactory for analysis: unlabeled or mislabeled samples, identification mismatch between specimen and requisition slip, haemolysed or clotted samples, wrong

For peer review only

collection tube, insufficient specimen quantity, excessive delay in specimen transport or improperly transported (i.e. not at required temperature).

Stool Sample:

Testing of stools for the isolation of poliovirus would be done according to the standardized protocol used at the Global Polio Laboratory Network (8, 9). Two stool specimens from the enrolled suspected primary immunodeficient children will be collected in sufficient quantity (~10 grams) 24 hours apart. Then the samples will be shipped to WHO Regional Reference Laboratory (RRL) for Polio Eradication Initiative (PEI), Virology Department, National Institute of Health (NIH)-Islamabad, Pakistan within 48 hours maintaining reverse cold chain along with patient's investigation questionnaire (also known as Lab request form). The lab request form indicated all general information such as demographic details (patient's name, age, sex, and region), vaccination history and clinical information. Each sample will be provided a unique identity code (specimen number).

Samples would then be tested for isolation of specific serotypes. Stool sample processing will start with the tissue culture in which specimen will be grown. Secondly, the molecular method will be used for the isolation and identification of poliovirus serotypes. Thirdly, two different assays of one-step real-time Reverse Transcriptase Polymerase Chain Reaction (rRT-PCR) will be used for detection of polioviruses using the protocols and primers/probes corresponding to 5′ UTR and VP1 region. These assays will include Intra-typic differentiation rRT-PCR that is for the confirmation, serotype identification and characterization for polioviruses. However, Reverse Transcriptase Real-Time rRT-PCR will be used for VDPV screening for vaccine-derived polioviruses screening. Testing would be done according to the WHO guidelines for the identification of poliovirus

Data Management and quality assurance:

Data Monitoring:

Regular supervision for the identification of PID children at the selected surveillance hospital all over Pakistan will be done by the iVDPV working group members-WGM. From each hospital, there would be two designated members who would be comprehensively trained on Jeffrey Modell Signs and identification of primary immunodeficiency by the study principal investigator. These members will be held responsible for correct identification of JMF signs, reviewing the filled surveillance questionnaire to ensure that it is filled properly and training the post-graduate students, residents and fellows on fundamentals of the project. Moreover, the project Principal Investigator (PI) and Research Coordinator (RC) of the study will also review forms and make visits to monitor the surveillance activity and to guide the working group members and whenever required. This iVDPV-WGM will also update RC about the daily progress of the study via phone calls or by sending a medical history summary report of suspected PIDs on the WhatsApp group generated for this study.

Quality of data collection will be ensured by proper training of the WGM. Quality will also be ensured through supervision and reviewing of the data collected by the principal investigator. Moreover,

For peer review only - http://bmjopen.bmj.com/site/about/guidelines.xhtml

skype/zoom meetings will be arranged to maintain coordination among the members of the team and to keep the members up-to-date and to resolve any coordination related issues.

Data Editing:

Data editing will be done in two phases: field site editing and office-based editing. Field editing will be done by the designated member of the surveillance site where they will check each questionnaire filled by attending physician on the hard copy of the questionnaire at the time of enrollment. These members will send the questionnaire to study RC for quality control and would be responsible for reviewing/editing if required at the central office-Aga Khan University, Karachi-Pakistan campus.

Data entry and cleaning:

Two data entry operators will enter the data into a data entry application in parallel. Data entered will be compared for entry errors and the identified errors will be corrected. The hard copies of surveillance questionnaire will be kept at the secure location until the project is being completed.

Plan of Analysis and sample size

Data will be analyzed using STATA version 11. Descriptive data analysis will be performed by calculating frequency with percentages for categorical data and mean with standard deviation or median with interquartile ranges for continuous scale data. We will calculate hospital-based proportions of total iVDPV cases in two years period. For sensitivity and specificity of the JMF signs, we will calculate receiver operating characteristics (ROC) curve by comparing the results based on screening with JMF signs with the results of Flow Cytometry and QIG tests. Since this is a surveillance, sample size calculation in not obligatory. However, for the sensitivity and specificity of JMF signs a minimum sample size of 50 PID cases, with 5% level of significance, change in sensitivity from 0.5 to 0.7 using two-sided binomial test and 78% power to detect a change in specificity from 0.5 to 0.7 using two-sided binomial test (7).

Limitations and strategies to overcome:

As PID is a rare condition and many children die before formal diagnosis, we may not be able to achieve the required sample size of 50 PID cases in two years duration. While the minimum sample size is not a requirement for the surveillance objective, it is needed for the calculation of sensitivity and specificity of the JMF 10 warning signs. To achieve sufficient sample size, the PI of the project will review the enrollment data quarterly and may decide to expand the sentinel sites to other tertiary care hospitals centers in the country to increase the number of enrollments.

Ethical Considerations

Ethical Approval

For peer review only

The surveillance project has been initiated after the formal ethical approval granted by the World Health Organization, Aga Khan University, National Bioethics Committee and Departmental Heads of Paediatrics from each participatory hospital.

Informed Consent

The formal written consent is taken from the parents or guardians of children with PID before the enrollment for blood and stool samples. Study details and processes are explained in detail, thumb impression is taken instead of a signature for parents who would be unable to read and write. Furthermore, it is recognized that no additional risk is involved as the blood collection for diagnostic testing is standard of care, and the stool collection poses no risks.

For HIV testing, we offer pre and post-test counselling to the families. If the test comes out positive, a referral will be made to the nearest National Aids Control program for free treatment and follow-ups.

Privacy and Confidentiality

The information from the participants will be taken by maintaining privacy and confidentiality. A unique ID will be issued to each participant based on the province and hospital so that the names of any participant might not be disclosed. For maintaining participant's confidentiality, all data forms, reports, and other records will be identified by the same unique identifier. All records will be kept in a secure location and will be shared only with those who will be directly involved in the study such as PI and the coordination team members at AKU and WHO. All the computerized entry will be done with unique ID's only.

Timeframe

This is a two-year project to commence after approval of ERC.

Dissemination Plan

The scientific paper resulting from this work will be submitted for the publication to peer-reviewed journal. This is the main study protocol paper which serves to inform about the design, methodology and coordination of the project. Once the surveillance is completed, study results will be published as a separate scientific paper. Abstracts would be submitted at relevant conferences, if selected will be presented in the form of poster or presentation. Study participants will be informed about the stool sample results as soon as it is available and shared by the NIH. In case of positive results for VDPV, parents will be told about further steps. A dissemination seminar will be conducted for the members of iVDPV working group, World Health Organization, policymakers, Paediatric consultants and fellow researchers with same domain interest.

For peer review only

Way Forward

Pakistan National Registry of Primary Immunodeficiency disease will be developed. The aim of the national PID registry will be to establish the epidemiological data of primary immunodeficiency. Moreover, the registry would serve as a database for the coordination cells on PID in Pakistan.

Authors' contributions:

AFS has developed the research question, conceptualized the study and led the group that secured funding. ***MO*** has an advisory role for study conceptualization and execution. ***MTY*** advised on methods, sample size calculations and statistical analysis. ***UN and MSR*** helped in liaison and support for iVDPV Surveillance in Pakistan. ***AP*** and ***ZK*** is responsible for study management and coordination and drafted the manuscript along with ***AFS*** and ***MO***. All authors have read, commented on and approved the final manuscript.

Funding statement: This work is supported by [World Health Organization], grant number [2018/811124-0].

Acknowledgement: The authors would like to extend their gratitude to Dr Priya Sukhtankar for English proofreading and editing. She is affiliated with Department of Paediatrics, Gloucestershire Royal Hospital NHS Foundation Trust.

References:

1. Li L, Ivanova O, Driss N, Tiongco-Recto M, Da Silva R, Shahmahmoodi S, et al. Poliovirus excretion among persons with primary immune deficiency disorders: summary of a seven-country study series. The Journal of infectious diseases. 2014;210(suppl_1):S368-S72.
2. Macklin G, Liao Y, Takane M, Dooling K, Gilmour S, Mach O, et al. Prolonged excretion of poliovirus among individuals with primary immunodeficiency disorder: an analysis of the World Health Organization registry. Frontiers in immunology. 2017;8:1103.
3. Organization WH, Initiative GPE. Polio endgame strategy 2019-2023: eradication, integration, certification and containment. World Health Organization; 2019.
4. Tebbens RJD, Pallansch MA, Thompson KM. Modeling the prevalence of immunodeficiency-associated long-term vaccine-derived poliovirus excretors and the potential benefits of antiviral drugs. BMC infectious diseases. 2015;15(1):379.
5. El-Sayed Z, Mach O, Hossny E, Galal N, El-Sawy I. Poliovirus Excretion among Persons with Primary Immune Deficiency Disorders: Summary of Data from Enhanced Poliovirus Surveillance in Egypt, 2011-2014. J Vaccines Vaccin 7: 331. doi: 10.4172/2157-7560.1000331 Page 2 of 6 J Vaccines Vaccin ISSN: 2157-7560 JVV, an open access journal Volume 7• Issue 4• 1000331. Characteristic n/N (%) N. 2016;122:0-1.
6. Sazzad HM, Rainey JJ, Kahn A-L, Mach O, Liyanage JB, Alam AN, et al. Screening for long-term poliovirus excretion among children with primary immunodeficiency disorders: preparation for the polio posteradication era in Bangladesh. The Journal of infectious diseases. 2014;210(suppl_1):S373-S9.

7. O'Sullivan MD, Cant AJ. The 10 warning signs: a time for a change? Current opinion in allergy and clinical immunology. 2012;12(6):588-94.
8. Hull BP, Dowdle WR. Poliovirus surveillance: building the global Polio Laboratory Network. The Journal of infectious diseases. 1997;175(Supplement_1):S113-S6.
9. Organization WH. Polio laboratory manual. Geneva: World Health Organization; 2004.

Figure 1- Participatory Tertiary care Hospitals

Figure 2- Flow diagram of the study process

For peer review only

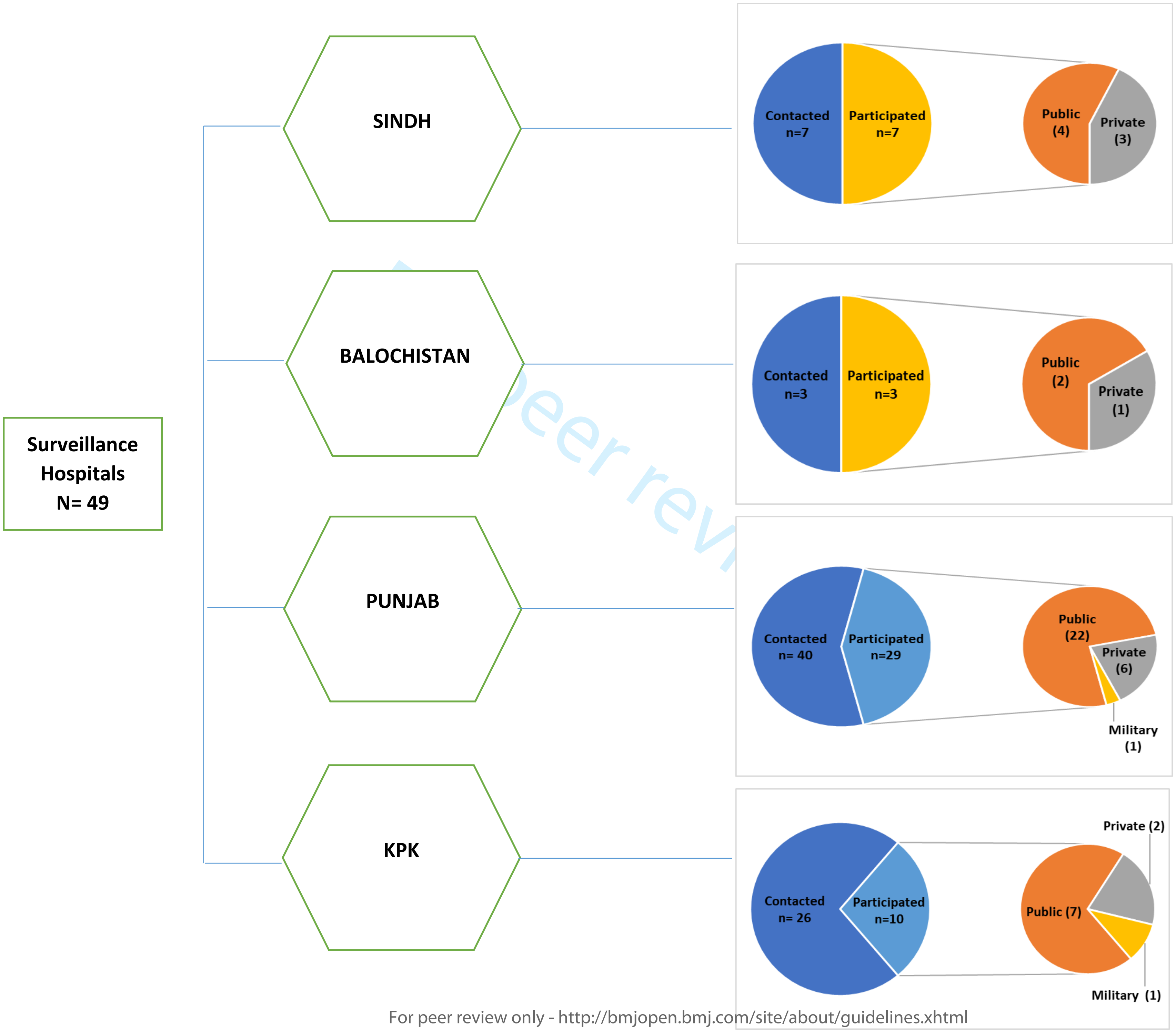

Figure 1- Participatory Tertiary care Hospitals

For peer review only

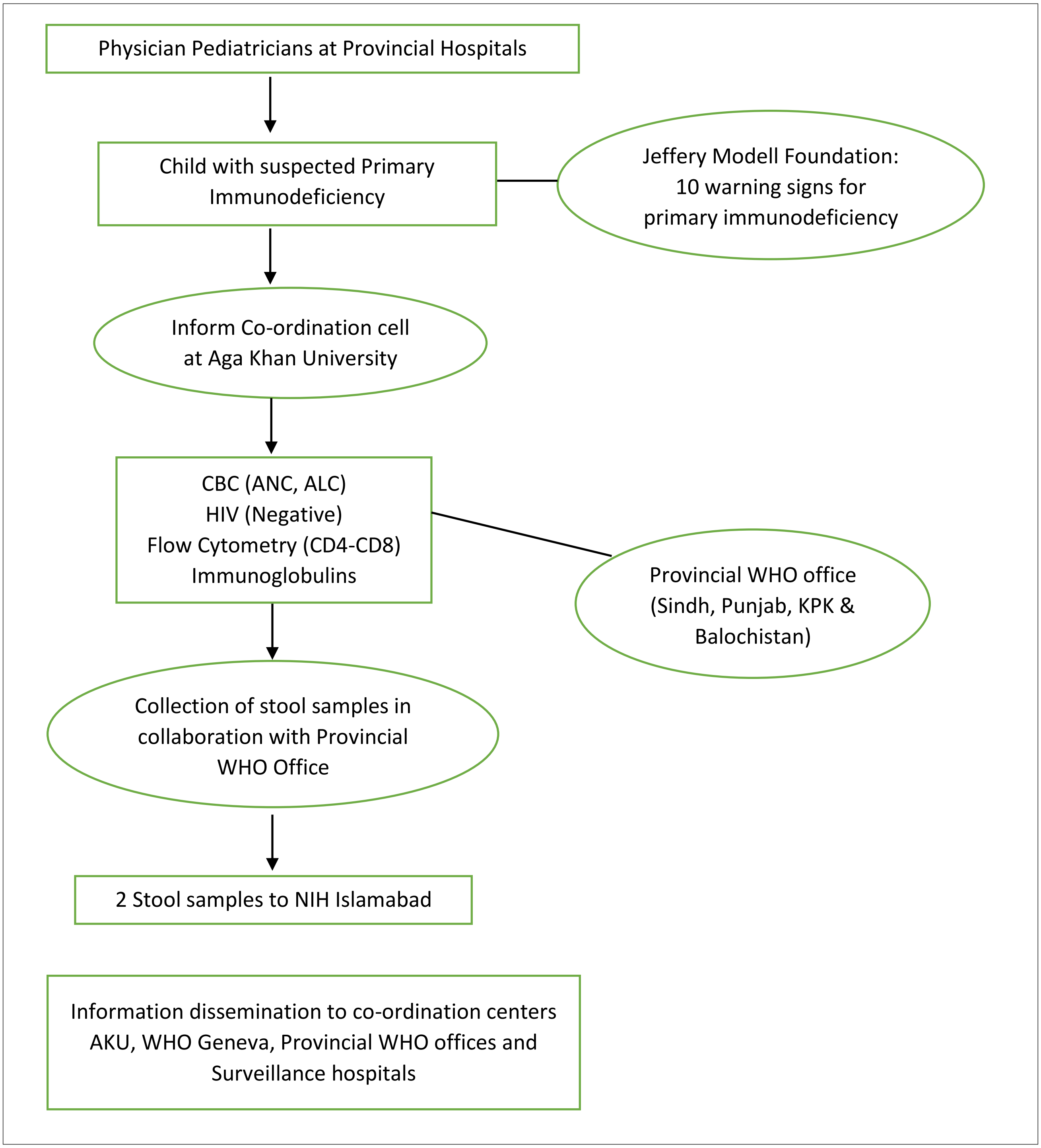

Figure 2- Flow diagram of study process

BMJ Open

Poliovirus Excretion Among Children with Primary Immune Deficiency in Pakistan- A Pilot Surveillance Study Protocol

Journal:	*BMJ Open*
Manuscript ID	bmjopen-2020-045904.R1
Article Type:	Protocol
Date Submitted by the Author:	26-Mar-2021
Complete List of Authors:	Pethani, Asma; Aga Khan University Hospital, Pediatrics and Child Health<br>Kazi, Zaubina; Aga Khan University, Pediatrics and Child Health<br>Nayyar, Ujala; World Health Organization Country Office for Pakistan, Polio Eradication Initiative<br>Shafiq-ur-Rehman, Muhammad ; World Health Organization Country Office for Pakistan<br>Yousafzai, Muhammad Tahir; Aga Khan University, Pediatrics and Child Health<br>Ondrej, Mach; World Health Organization<br>Saleem, Ali; Aga Khan University, Paediatrics and Child Health
**Primary Subject Heading**:	Paediatrics
Secondary Subject Heading:	Infectious diseases, Immunology (including allergy), Public health
Keywords:	INFECTIOUS DISEASES, PAEDIATRICS, Public health < INFECTIOUS DISEASES

SCHOLARONE™
Manuscripts

I, the Submitting Author has the right to grant and does grant on behalf of all authors of the Work (as defined in the below author licence), an exclusive licence and/or a non-exclusive licence for contributions from authors who are: i) UK Crown employees; ii) where BMJ has agreed a CC-BY licence shall apply, and/or iii) in accordance with the terms applicable for US Federal Government officers or employees acting as part of their official duties; on a worldwide, perpetual, irrevocable, royalty-free basis to BMJ Publishing Group Ltd ("BMJ") its licensees and where the relevant Journal is co-owned by BMJ to the co-owners of the Journal, to publish the Work in this journal and any other BMJ products and to exploit all rights, as set out in our licence.

The Submitting Author accepts and understands that any supply made under these terms is made by BMJ to the Submitting Author unless you are acting as an employee on behalf of your employer or a postgraduate student of an affiliated institution which is paying any applicable article publishing charge ("APC") for Open Access articles. Where the Submitting Author wishes to make the Work available on an Open Access basis (and intends to pay the relevant APC), the terms of reuse of such Open Access shall be governed by a Creative Commons licence – details of these licences and which Creative Commons licence will apply to this Work are set out in our licence referred to above.

Other than as permitted in any relevant BMJ Author's Self Archiving Policies, I confirm this Work has not been accepted for publication elsewhere, is not being considered for publication elsewhere and does not duplicate material already published. I confirm all authors consent to publication of this Work and authorise the granting of this licence.

For peer review only

Poliovirus Excretion among Children with Primary Immune Deficiency in Pakistan, a Pilot Surveillance Study Protocol

Asma Pethani[1], Zaubina Kazi[1], Ujala Nayyar[2], Muhammad Shafiq-ur-Rehman[3], Muhammad Tahir Yousafzai[1], Mach Ondrej[4], Ali Faisal Saleem[1*]

[1]Department of Paediatrics & Child Health, Aga Khan University, Karachi

[2]Polio Eradication Initiative, World Health Organization, Pakistan

[3]World Health Organization and National Emergency Operations Centre, Pakistan

[4]World Health Organization, Geneva, Switzerland

*Corresponding author, ali.saleem@aku.edu

Abstract

Introduction:

Children with primary immunodeficiency disorders (PID) are more susceptible to developing viral infections and are at a substantially increased risk of developing paralytic poliomyelitis. Such children, if given oral polio vaccines tend to excrete poliovirus chronically that may lead to the propagation of highly divergent vaccine-derived poliovirus (VDPVs). Consequently, they may act as a reservoir for the community by introducing an altered virus potentially imposing a risk to global polio eradication. However, the risks of chronic and prolonged excretion are not well characterized in the study context. This study seeks to establish a pilot surveillance system for successful identification and monitoring of vaccine-derived poliovirus excretion among children with PID. It will assess whether the Jeffrey Modell warning signs of PID can be utilized as an appropriate screening tool for PID in Pakistan.

Methods and Analysis:

In this pilot surveillance, recruitment of PID cases is currently done at participating hospitals in Pakistan. Potential children are screened and tested against the Jeffrey Modell Foundation (JMF) warning signs for immunodeficiency and their stool is collected to test for poliovirus excretion. Cases excreting poliovirus are followed until the two consecutive negative stool samples are obtained over a period of six months. The data will be analyzed to calculate hospital-based proportions of total iVDPV cases over a two-year period and to determine the sensitivity and specificity of the JMF signs.

Ethics and Dissemination:

This protocol was reviewed and approved by the World Health Organization (WHO Reference-2018/811124-0), Aga Khan University (AKU ERC- 2018-0380-1029) and National Bioethics Committee (Ref No. 4-87 NBC-308-Y2). The results will be published in an open access peer-reviewed scientific journal and presented to the iVDPV working-group members, policy-makers,

For peer review only

paediatric consultants and fellow researchers with the same domain interest. It may be presented in scientific conferences and seminars in the form of oral or poster presentations.

Strengths and limitations:

- PID patients are screened for excretion of poliovirus for the first time in Pakistan
- Jeffrey Modell warning signs for PID are used together with immunological diagnostic testing for PID
- Poliovirus excretors are followed up in monthly intervals until two consecutive samples are negative
- Proportions and types of poliovirus excretors among PID patients are analyzed monthly
- While attempts were made to cover entire Pakistan, the screening may miss PID cases in more remote areas

For peer review only

Introduction

Polio is a highly infectious viral disease which invades the nervous system of a child and may cause irreversible paralysis (1). The use of the live oral poliovirus vaccine (OPV) in routine and supplementary immunization activities (SIAs) has significantly reduced the incidence of paralytic poliomyelitis and transmission of wild polioviruses around the globe. This success can be attributed to the ability of this vaccine to induce mucosal immunity as well as serum neutralizing antibodies protective against paralytic poliomyelitis (2). However, individuals with primary immune deficiency disorders (PID) exposed to OPV through vaccination or household contacts are not only at increased risk of vaccine-associated paralytic poliomyelitis (VAPP) but may also have prolonged excretion of Sabin vaccine-derived polioviruses (VDPVs) (3). Sabin polioviruses are known to have increased transmissibility and neurovirulence following prolonged intestinal replication. Such individuals could theoretically transmit the virus to contacts and the general population following the interruption of wild poliovirus transmission (4). These polioviruses are called iVDPVs, which indicates that these viruses have replicated and evolved in immuno-compromised individuals. Previous studies in Sri Lanka, Egypt, Bangladesh and elsewhere demonstrated that it is essential to identify PID persons excreting polioviruses (1, 5, 6).

Poliovirus excretion into the environment negates global poliovirus eradication efforts. PID individuals infected with poliovirus are at risk of developing paralysis. Antiviral therapy will be offered to those identified immunodeficient children who are excreting polioviruses to prevent the spread of these polioviruses.

Aim:

Identification and diagnosis of PID is complex and requires specialist interventions. The Jeffrey Modell Foundation has developed 10 warning signs for the screening of suspected primary immunodeficiency patients (7). Although this screening tool has been used in various countries, its sensitivity and specificity in low-resource countries such as Pakistan has not been assessed. The primary aim of this project is to develop a pilot surveillance system for the identification of primary immunodeficient children in sentinel sites of Pakistan which will provide the basis for the development of longer-term surveillance structure for the successful identification, genetic characterization and monitoring of vaccine-derived poliovirus excretion. This will help in identifying long-term poliovirus excreters among them, which will in return help in the prevention of future transmission and VDPV outbreaks.

Secondary aims will be to calculate the hospital-based proportion of iVDPV cases and to document the identified iVDPV cases in the WHO global registry list. Finally, it will help assess whether the Jeffrey Modell warning signs of PID are an appropriate screening tool for PID in Pakistan.

Methodology

Project Structure:

For peer review only - http://bmjopen.bmj.com/site/about/guidelines.xhtml

The surveillance of iVDPV is currently being undertaken at participatory tertiary healthcare facilities around Pakistan. This pilot surveillance will help in structuring a sustainable, long-term surveillance for PID children in Pakistan. In the initial stage, mapping was executed to identify participatory hospitals and their Consultant Pediatricians. Master training workshops were then conducted for these paediatricians from the selected hospitals of each province of Pakistan who agreed to participate in iVDPV surveillance. The project was rolled out as follows:

- First 6 months: AKU Hospital + 3 other major tertiary care hospitals in Karachi
- Months 7-24: AKU Hospital + Major hospitals in Karachi + Tertiary hospitals located in the provinces of Sindh (Hyderabad), Punjab (Lahore, Multan, Islamabad), Balochistan (Quetta), Khyber Pakhtunkhwa and Gilgit-Baltistan.

Training of Master Trainers Workshop:

The master training workshops were conducted all over the country in collaboration with World Health Organization, Pakistan. All tertiary care hospitals in each province with paediatricians were approached. Across Pakistan, 49 tertiary care hospitals have been participating in iVDPV surveillance. The participatory rate from the province Sindh (n=7) and Balochistan (n=3) is 100%, followed by 72.5% (n=29) from the province Punjab. The participatory proportion from the province Khyber Pakhtunkhwa (n=10) is 38.5%, which is the lowest amongst the remaining provinces **(Refer to *Figure 1*).** The purpose of the training workshop was to train the master trainers who in turn guided their departmental doctors for screening and identification of PID children using the JMF warning signs.

The master training workshops were conducted by the principal investigator of this project who is a pediatric infectious diseases consultant at the Aga Khan University Hospital in collaboration with WHO experts in Polio Surveillance.

Co-ordination Process:

This study requires close collaboration and coordination amongst the stakeholders. The Aga Khan University Hospital (Pakistan) is the main coordination center for the study. A Research Coordinator serves as a liaison between the participatory hospitals in each province and the WHO provincial team, which collects the stool samples of identified PID children. National Institute of Health, Islamabad virology lab processes the stool samples and identifies the type of poliovirus.

Data Collection Process:

Children are identified by the paediatricians trained on Jeffrey Model Foundation warning signs and the diagnosis of primary immunodeficiency. Any child (up to 18 years of age) fulfilling the criteria of 2 or more positive JMF signs, will be further assessed and tested for HIV. The children who test negative for HIV, either suspected or confirmed PIDs are considered potential iVDPV excreters and are enrolled in the study. Enrolled children will be further tested for complete blood count (CBC), flow cytometry and quantitative measurement of immunoglobulins level (QIGs) after consulting with

For peer review only

the co-ordination cell at the Aga Khan University. A consultant haematologist at the Aga Khan University will review the flow cytometry and immunoglobulins level reports. After enrollment, WHO provincial teams collect two consecutive stool samples, which are examined for poliovirus at NIH Islamabad-Pakistan using standard poliovirus detection methodology used in the Global Polio Laboratory Network. The second stool sample will be collected 24 hours after the first sample. Any child found to be positive will be followed-up with monthly stool samples for 6 months or till two subsequent samples become negative. All iVDPVs detected will be documented in the WHO global registry list of known iVDPV cases.

Follow-up with the iVDPV cases:

The parents or guardians of iVDPV positive children are informed and followed up closely by EPI and Union Council teams along with WHO provincial teams. Children are followed up until two consecutive negative stool samples for a period of six months. However, if the child is still found to be an excreters at 6 months, they will be provided anti-viral therapy for poliovirus excretion. This therapy would be provided to the affected child free of cost.

The children who test negative for VDPVs or Sabin like virus, follow-up stool sample collection will be after a year.

Process:

In order to maximize the surveillance coverage of primary immune deficient children, hospital pediatricians will be sufficiently sensitized to the necessary referral process.

Children with suspected PID screened using JMF warning signs will be enrolled and the following laboratory investigations would be sent for confirmation ***(Refer to Figure 2- Flow diagram of study process).***

1. Complete blood count (Absolute neutrophil and lymphocyte count)
2. HIV
3. Flow cytometry (B and T cell markers)
4. Quantitative immunoglobulins test (QIG)

Patient and Public Involvement:

Considering the nature of the study, it was not possible to have a priori patient involvement. The country leadership from World Health Organization and National Emergency Operations Centre (NEOC) were consulted during the study designing to safeguard public interest and safety.

Biological sample collection, transport and laboratory processing:

Blood Samples:

For peer review only

HIV:

For HIV testing, 3-5 ml of whole blood will be collected in a gel tube and K2-EDTA tube. Gel tube will be utilized for serology whereas, EDTA tube would use for PCR. Samples will be transported to the laboratory at 2-8 ^{0}C. Initial HIV screening will be performed by chemiluminescent immunoassay (CLIA) using ADVIA Centaur HIV Ag/Ab Combo assay kit run on ADVIA Centaur XP/CP analyzer. Positive cases will be further confirmed by immunochromatographic antibody detection on Bio-Rad Genius HIV 1 / 2 assay. In case of indeterminate results or discrepant results between the above two methods final confirmation will be made by HIV PCR on Xpert HIV-1 Qual Assay kit and GeneXpert Dx System.

CBC

For complete blood count, 3-4ml whole blood sample will be collected in tubes containing K2 EDTA as an anticoagulant. Samples will be transported as soon as possible to the stat/main laboratory. The temperature of 18 –25 ^{0}C will be maintained if the expected delivery time is within 8 hours, whereas 2 – 8 ^{0}C will be maintained if samples are delivered after 8 hours up to a maximum of 3 days. Samples will be run on the fully automated Sysmex XN series analyzers (Sysmex Japan).

Immunoglobulins

For immunoglobulin measurement, 3-5 ml of whole blood will be collected in a plain tube or gel tube. Transportation temperature of 18-25°C (for 8 Hours) and 2-8 °C after 8 hours to 24 hours will be maintained. Serum Immunoglobulin A, G and M (IgA, IgG, IgM) will be measured on ADVIA 1800 Chemistry analyzer (Siemens Healthcare Diagnostics Inc, NY, US).

Flow Cytometry

For lymphocyte subset analysis, 3-4ml of whole blood will be collected in tubes containing K2 EDTA or sodium heparin as an anticoagulant. All samples will be transported at 2-8^{0}C within 24 hours. Lymphocyte subset analysis will be performed by flow cytometry using FC500 MCL Flow Cytometer (Beckman Coulter, Miami, FL, USA). Monoclonal antibodies against following antigens were used: T-lineage-associated antigens CD3, CD4 and CD8; B-lineage antigen CD19 and Natural Killer cell antigen CD56.

For CBC and immunoglobulin levels, three different levels of manufacturer-provided controls (low, normal and high) will be run with each batch as an internal quality control measure. Whereas, for lymphocyte subset analysis 2 levels (low and normal) commercial controls will be used. External proficiency is assured by simultaneously analyzing samples from the College of American Pathologists 2 times per year for immunoglobulin levels and three times per year for both complete blood counts and lymphocyte subset analysis.

For peer review only

Following samples will be considered unsatisfactory for analysis: unlabeled or mislabeled samples, identification mismatch between specimen and requisition slip, haemolysed or clotted samples, wrong collection tube, insufficient specimen quantity, excessive delay in specimen transport or improperly transported (i.e. not at required temperature).

Stool Sample:

Testing of stools for the isolation of poliovirus would be done according to the standardized protocol used at the Global Polio Laboratory Network (8, 9). Two stool specimens from the enrolled suspected primary immunodeficient children will be collected in sufficient quantity (~10 grams) 24 hours apart. Then the samples will be shipped to WHO Regional Reference Laboratory (RRL) for Polio Eradication Initiative (PEI), Virology Department, National Institute of Health (NIH)-Islamabad, Pakistan within 48 hours maintaining reverse cold chain along with patient's investigation questionnaire (also known as Lab request form). The lab request form indicated all general information such as demographic details (patient's name, age, sex, and region), vaccination history and clinical information. Each sample will be provided a unique identity code (specimen number).

Samples will then be tested for isolation of specific serotypes. Stool sample processing will start with the tissue culture in which specimen will be grown. Secondly, the molecular method will be used for the isolation and identification of poliovirus serotypes. Thirdly, two different assays of one-step real-time Reverse Transcriptase Polymerase Chain Reaction (rRT-PCR) will be used for detection of polioviruses using the protocols and primers/probes corresponding to 5′ UTR and VP1 region. These assays will include Intra-typic differentiation rRT-PCR that is for the confirmation, serotype identification and characterization for polioviruses. However, Reverse Transcriptase Real-Time rRT-PCR will be used for VDPV screening for vaccine-derived polioviruses screening. Testing will be done according to the WHO guidelines for the identification of poliovirus

Data Management and quality assurance:

Data Monitoring:

Regular supervision for the identification of PID children at the selected surveillance hospital all over Pakistan will be done by the iVDPV working group members-WGM. From each hospital, there would be two designated members who would be comprehensively trained on Jeffrey Modell Signs and identification of primary immunodeficiency by the study principal investigator. These members will be held responsible for correct identification of JMF signs, reviewing the filled surveillance questionnaire to ensure that it is filled properly and training the post-graduate students, residents and fellows on fundamentals of the project. Moreover, the project Principal Investigator (PI) and Research Coordinator (RC) of the study will also review forms and make visits to monitor the surveillance activity and to guide the working group members and whenever required. This iVDPV-WGM will also update RC about the daily progress of the study via phone calls or by sending a medical history summary report of suspected PIDs on the WhatsApp group generated for this study.

For peer review only

Quality of data collection will be ensured by proper training of the WGM. Quality will also be ensured through supervision and reviewing of the data collected by the principal investigator. Moreover, skype/zoom meetings will be arranged to maintain coordination among the members of the team and to keep the members up-to-date and to resolve any coordination related issues.

Data Editing:

Data editing will be done in two phases: field site editing and office-based editing. Field editing will be done by the designated member of the surveillance site where they will check each questionnaire filled by attending physician on the hard copy of the questionnaire at the time of enrollment. These members will send the questionnaire to study RC for quality control and would be responsible for reviewing/editing if required at the central office-Aga Khan University, Karachi-Pakistan campus.

Data entry and cleaning:

Two data entry operators will enter the data into a data entry application in parallel. Data entered will be compared for entry errors and the identified errors will be corrected. The hard copies of surveillance questionnaire will be kept at the secure location until the project is being completed.

Plan of Analysis and sample size

Data will be analyzed using STATA version 11. Descriptive data analysis will be performed by calculating frequency with percentages for categorical data and mean with standard deviation or median with interquartile ranges for continuous scale data. We will calculate hospital-based proportions of total iVDPV cases in two years period. For sensitivity and specificity of the JMF signs, we will calculate receiver operating characteristics (ROC) curve by comparing the results based on screening with JMF signs with the results of Flow Cytometry and QIG tests. Since this is a surveillance, sample size calculation in not obligatory. However, for the sensitivity and specificity of JMF signs a minimum sample size of 50 PID cases, with 5% level of significance, change in sensitivity from 0.5 to 0.7 using two-sided binomial test and 78% power to detect a change in specificity from 0.5 to 0.7 using two-sided binomial test (7).

Limitations and strategies to overcome:

As PID is a rare condition and many children die before formal diagnosis, we may not be able to achieve the required sample size of 50 PID cases in two years duration. While the minimum sample size is not a requirement for the surveillance objective, it is needed for the calculation of sensitivity and specificity of the JMF 10 warning signs. To achieve sufficient sample size, the PI of the project will review the enrollment data quarterly and may decide to expand the sentinel sites to other tertiary care hospitals centers in the country to increase the number of enrollments.

Ethics and dissemination

For peer review only

Ethical Approval

The surveillance project has been initiated after the formal ethical approval granted by the World Health Organization, Aga Khan University, National Bioethics Committee and Departmental Heads of Paediatrics from each participatory hospital.

Informed Consent

The formal written consent is taken from the parents or guardians of children with PID before the enrollment for blood and stool samples. Study details and processes are explained in detail, thumb impression is taken instead of a signature for parents who would be unable to read and write. Furthermore, it is recognized that no additional risk is involved as the blood collection for diagnostic testing is standard of care, and the stool collection poses no risks.

For HIV testing, we offer pre and post-test counselling to the families. If the test comes out positive, a referral will be made to the nearest National Aids Control program for free treatment and follow-ups.

Privacy and Confidentiality

The information from the participants will be taken by maintaining privacy and confidentiality. A unique ID will be issued to each participant based on the province and hospital so that the names of any participant might not be disclosed. For maintaining participant's confidentiality, all data forms, reports, and other records will be identified by the same unique identifier. All records will be kept in a secure location and will be shared only with those who will be directly involved in the study such as PI and the coordination team members at AKU and WHO. All the computerized entry will be done with unique ID's only.

Timeframe

This is a two-year project to commence after approval of ERC.

Dissemination Plan

The scientific paper resulting from this work will be submitted for the publication to peer-reviewed journal. This is the main study protocol paper which serves to inform about the design, methodology and coordination of the project. Once the surveillance is completed, study results will be published as a separate scientific paper. Abstracts would be submitted at relevant conferences, if selected will be presented in the form of poster or presentation. Study participants will be informed about the stool sample results as soon as it is available and shared by the NIH. In case of positive results for VDPV, parents will be told about further steps. A dissemination seminar will be conducted for the members of iVDPV working group, World Health Organization, policymakers, Paediatric consultants and fellow researchers with same domain interest.

For peer review only

Way Forward

Pakistan National Registry of Primary Immunodeficiency disease will be developed. The aim of the national PID registry will be to establish the epidemiological data of primary immunodeficiency. Moreover, the registry would serve as a database for the coordination cells on PID in Pakistan.

Authors' contributions:

AFS has developed the research question, conceptualized the study and led the group that secured funding. ***MO*** has an advisory role for study conceptualization and execution. ***MTY*** advised on methods, sample size calculations and statistical analysis. ***UN and MSR*** helped in liaison and support for iVDPV Surveillance in Pakistan. ***AP*** and ***ZK*** is responsible for study management and coordination and drafted the manuscript along with ***AFS*** and ***MO***. All authors have read, commented on and approved the final manuscript.

Competing Interests: There are no competing interests for any author.

Funding statement: This work is supported by [World Health Organization], grant number [2018/811124-0].

Data sharing statement: No additional data available.

Acknowledgement: The authors would like to extend their gratitude to Dr Priya Sukhtankar for English proofreading and editing. She is affiliated with Department of Paediatrics, Gloucestershire Royal Hospital NHS Foundation Trust.

References:

1. Li L, Ivanova O, Driss N, Tiongco-Recto M, Da Silva R, Shahmahmoodi S, et al. Poliovirus excretion among persons with primary immune deficiency disorders: summary of a seven-country study series. The Journal of infectious diseases. 2014;210(suppl_1):S368-S72.
2. Macklin G, Liao Y, Takane M, Dooling K, Gilmour S, Mach O, et al. Prolonged excretion of poliovirus among individuals with primary immunodeficiency disorder: an analysis of the World Health Organization registry. Frontiers in immunology. 2017;8:1103.
3. Organization WH, Initiative GPE. Polio endgame strategy 2019-2023: eradication, integration, certification and containment. World Health Organization; 2019.
4. Tebbens RJD, Pallansch MA, Thompson KM. Modeling the prevalence of immunodeficiency-associated long-term vaccine-derived poliovirus excretors and the potential benefits of antiviral drugs. BMC infectious diseases. 2015;15(1):379.
5. El-Sayed Z, Mach O, Hossny E, Galal N, El-Sawy I. Poliovirus Excretion among Persons with Primary Immune Deficiency Disorders: Summary of Data from Enhanced Poliovirus Surveillance in Egypt, 2011-2014. J Vaccines Vaccin 7: 331. doi: 10.4172/2157-7560.1000331 Page 2 of 6 J Vaccines Vaccin ISSN: 2157-7560 JVV, an open access journal Volume 7• Issue 4• 1000331. Characteristic n/N (%) N. 2016;122:0-1.

For peer review only - http://bmjopen.bmj.com/site/about/guidelines.xhtml

6. Sazzad HM, Rainey JJ, Kahn A-L, Mach O, Liyanage JB, Alam AN, et al. Screening for long-term poliovirus excretion among children with primary immunodeficiency disorders: preparation for the polio posteradication era in Bangladesh. The Journal of infectious diseases. 2014;210(suppl_1):S373-S9.

7. O'Sullivan MD, Cant AJ. The 10 warning signs: a time for a change? Current opinion in allergy and clinical immunology. 2012;12(6):588-94.

8. Hull BP, Dowdle WR. Poliovirus surveillance: building the global Polio Laboratory Network. The Journal of infectious diseases. 1997;175(Supplement_1):S113-S6.

9. Organization WH. Polio laboratory manual. Geneva: World Health Organization; 2004.

Figure 1- Participatory Tertiary care Hospitals

Figure 2- Flow diagram of the study process

For peer review only

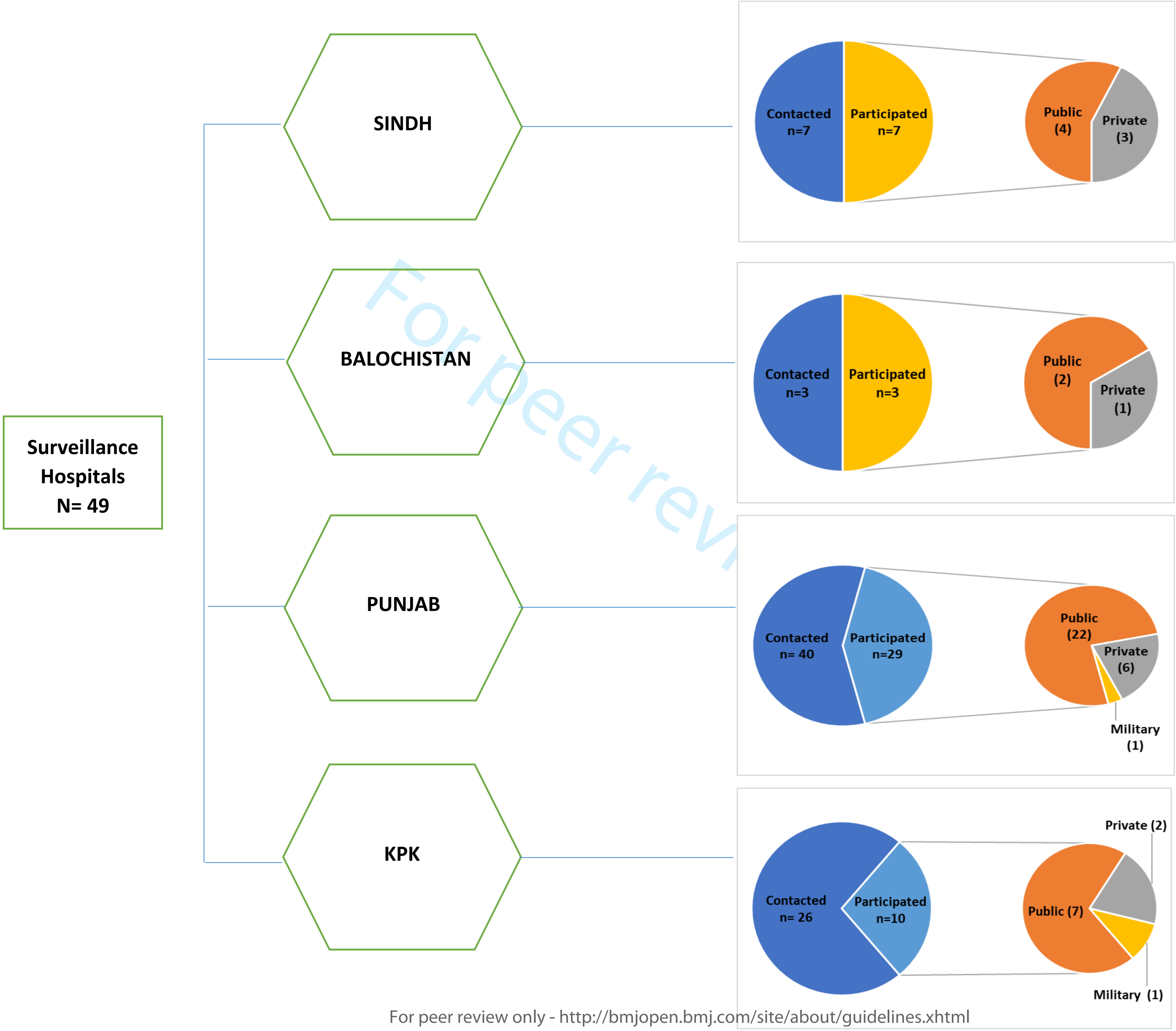

Figure 1- Participatory Tertiary care Hospitals

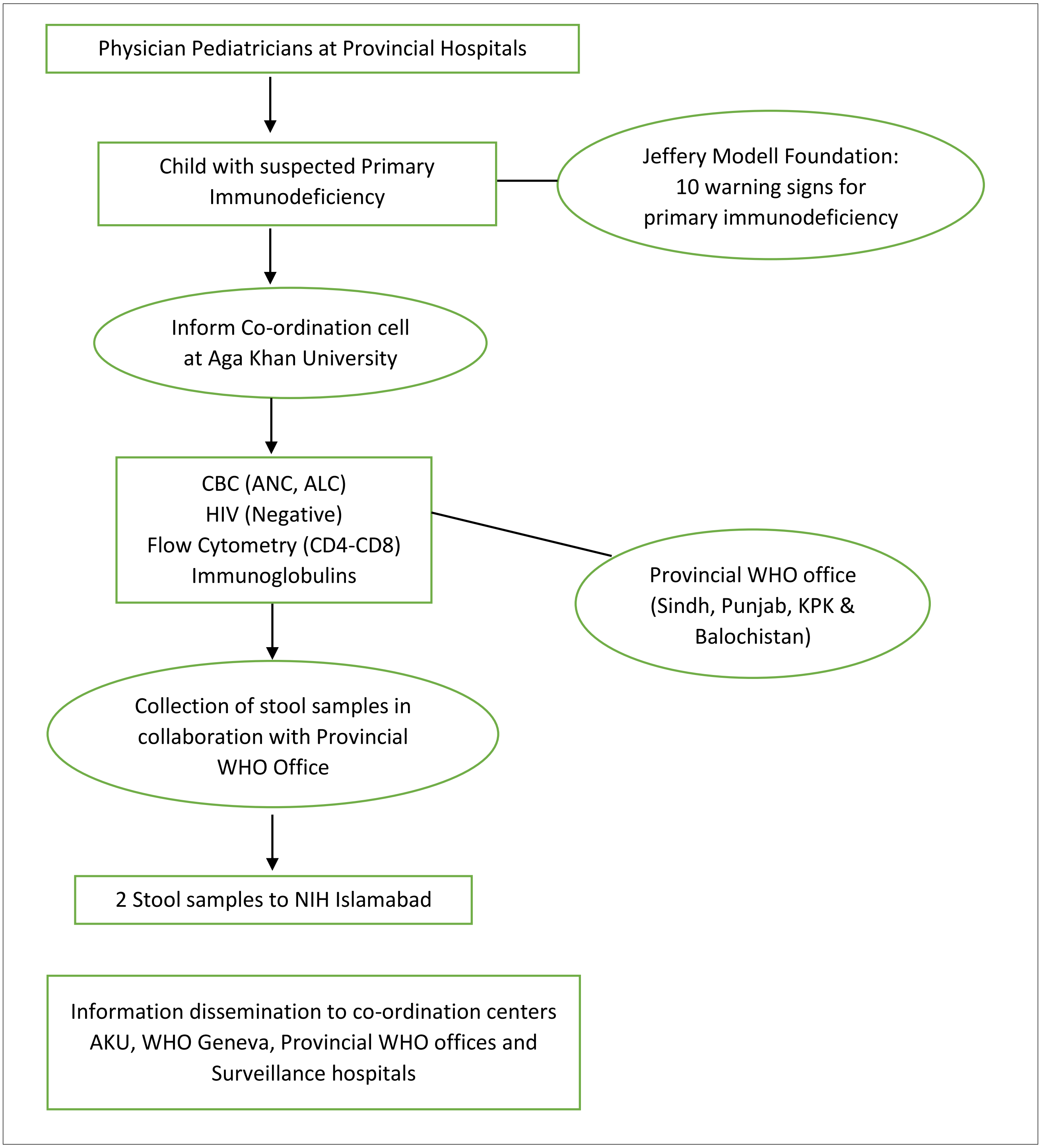

Figure 2- Flow diagram of study process

STROBE 2007 (v4) checklist of items to be included in reports of observational studies in epidemiology*
Checklist for cohort, case-control, and cross-sectional studies (combined)

Section/Topic	Item #	Recommendation	Reported on page #
**Title and abstract**	1	(*a*) Indicate the study's design with a commonly used term in the title or the abstract	1
		(*b*) Provide in the abstract an informative and balanced summary of what was done and what was found	1
**Introduction**			
Background/rationale	2	Explain the scientific background and rationale for the investigation being reported	3
Objectives	3	State specific objectives, including any pre-specified hypotheses	3
**Methods**			
Study design	4	Present key elements of study design early in the paper	4
Setting	5	Describe the setting, locations, and relevant dates, including periods of recruitment, exposure, follow-up, and data collection	4-5
Participants	6	(*a*) *Cohort study*—Give the eligibility criteria, and the sources and methods of selection of participants. Describe methods of follow-up<br>*Case-control study*—Give the eligibility criteria, and the sources and methods of case ascertainment and control selection. Give the rationale for the choice of cases and controls<br>*Cross-sectional study*—Give the eligibility criteria, and the sources and methods of selection of participants	5
		(*b*) *Cohort study*—For matched studies, give matching criteria and number of exposed and unexposed<br>*Case-control study*—For matched studies, give matching criteria and the number of controls per case	N/A
Variables	7	Clearly define all outcomes, exposures, predictors, potential confounders, and effect modifiers. Give diagnostic criteria, if applicable	5
Data sources/ measurement	8*	For each variable of interest, give sources of data and details of methods of assessment (measurement). Describe comparability of assessment methods if there is more than one group	N/A
Bias	9	Describe any efforts to address potential sources of bias	N/A
Study size	10	Explain how the study size was arrived at	8
Quantitative variables	11	Explain how quantitative variables were handled in the analyses. If applicable, describe which groupings were chosen and why	8 (Plan of Analysis)
Statistical methods	12	(*a*) Describe all statistical methods, including those used to control for confounding	N/A
		(*b*) Describe any methods used to examine subgroups and interactions	N/A
		(*c*) Explain how missing data were addressed	N/A
		(*d*) *Cohort study*—If applicable, explain how loss to follow-up was addressed<br>*Case-control study*—If applicable, explain how matching of cases and controls was addressed	N/A

For peer review only

		Cross-sectional study—If applicable, describe analytical methods taking account of sampling strategy	
		(*e*) Describe any sensitivity analyses	
**Results**			
Participants	13*	(a) Report numbers of individuals at each stage of study—eg numbers potentially eligible, examined for eligibility, confirmed eligible, included in the study, completing follow-up, and analysed	N/A
		(b) Give reasons for non-participation at each stage	
		(c) Consider use of a flow diagram	
Descriptive data	14*	(a) Give characteristics of study participants (eg demographic, clinical, social) and information on exposures and potential confounders	N/A
		(b) Indicate number of participants with missing data for each variable of interest	
		(c) *Cohort study*—Summarise follow-up time (eg, average and total amount)	
Outcome data	15*	*Cohort study*—Report numbers of outcome events or summary measures over time	N/A
		Case-control study—Report numbers in each exposure category, or summary measures of exposure	
		Cross-sectional study—Report numbers of outcome events or summary measures	
Main results	16	(*a*) Give unadjusted estimates and, if applicable, confounder-adjusted estimates and their precision (eg, 95% confidence interval). Make clear which confounders were adjusted for and why they were included	N/A
		(*b*) Report category boundaries when continuous variables were categorized	
		(*c*) If relevant, consider translating estimates of relative risk into absolute risk for a meaningful time period	
Other analyses	17	Report other analyses done—eg analyses of subgroups and interactions, and sensitivity analyses	N/A
**Discussion**			
Key results	18	Summarise key results with reference to study objectives	N/A
Limitations	19	Discuss limitations of the study, taking into account sources of potential bias or imprecision. Discuss both direction and magnitude of any potential bias	9
Interpretation	20	Give a cautious overall interpretation of results considering objectives, limitations, multiplicity of analyses, results from similar studies, and other relevant evidence	N/A
Generalisability	21	Discuss the generalisability (external validity) of the study results	N/A
**Other information**			
Funding	22	Give the source of funding and the role of the funders for the present study and, if applicable, for the original study on which the present article is based	10

*Give information separately for cases and controls in case-control studies and, if applicable, for exposed and unexposed groups in cohort and cross-sectional studies.

Note: An Explanation and Elaboration article discusses each checklist item and gives methodological background and published examples of transparent reporting. The STROBE checklist is best used in conjunction with this article (freely available on the Web sites of PLoS Medicine at http://www.plosmedicine.org/, Annals of Internal Medicine at http://www.annals.org/, and Epidemiology at http://www.epidem.com/). Information on the STROBE Initiative is available at www.strobe-statement.org.

For peer review only
